# RNA Aptamers as Molecular Tools to Study the Functionality of the Hepatitis C Virus CRE Region

**DOI:** 10.3390/molecules200916030

**Published:** 2015-09-02

**Authors:** Alba Fernández-Sanlés, Beatriz Berzal-Herranz, Rodrigo González-Matamala, Pablo Ríos-Marco, Cristina Romero-López, Alfredo Berzal-Herranz

**Affiliations:** Instituto de Parasitología y Biomedicina López-Neyra (IPBLN-CSIC), PTS Granada, Av. Conocimiento, 17, 18016 Armilla, Granada, Spain; E-Mails: alba.fernandez@upf.edu (A.F.-S.); bbh@ipb.csic.es (B.B.-H.); rurik.rgm@gmail.com (R.G.-M.); priosm@ipb.csic.es (P.R.-M.)

**Keywords:** RNA aptamers, antiHCV Aptamers, HCV genome, CRE, 5BSL3.2, functional RNA domains

## Abstract

Background: Hepatitis C virus (HCV) contains a (+) ssRNA genome with highly conserved structural, functional RNA domains, many of them with unknown roles for the consecution of the viral cycle. Such genomic domains are candidate therapeutic targets. This study reports the functional characterization of a set of aptamers targeting the *cis*-acting replication element (CRE) of the HCV genome, an essential partner for viral replication and also involved in the regulation of protein synthesis. Methods: Forty-four aptamers were tested for their ability to interfere with viral RNA synthesis in a subgenomic replicon system. Some of the most efficient inhibitors were further evaluated for their potential to affect the recruitment of the HCV RNA-dependent RNA polymerase (NS5B) and the viral translation in cell culture. Results: Four aptamers emerged as potent inhibitors of HCV replication by direct interaction with functional RNA domains of the CRE, yielding a decrease in the HCV RNA levels higher than 90%. Concomitantly, one of them also induced a significant increase in viral translation (>50%). The three remaining aptamers efficiently competed with the binding of the NS5B protein to the CRE. Conclusions: Present findings confirm the potential of the CRE as an anti-HCV target and support the use of aptamers as molecular tools for investigating the functionality of RNA domains in viral genomes.

## 1. Introduction

The hypothesis of an all-RNA based world inspired the recreation of the natural molecular selection and evolution processes in a test tube, with the aim of isolating nucleic acids with diverse activities. These efforts yielded the identification of the so-called aptamers, oligonucleotides able to recruit a wide variety of ligands [1,2]. Aptamers are isolated from a SELEX (Systematic Evolution of Ligands by Exponential enrichment) process, which consists on iterative sets of synthesis, binding, positive selection, and amplification steps over a randomized oligonucleotide pool. The resulting population is enriched in those molecules able to bind to the desired target molecule. The highly dynamic folding of nucleic acids is the key to understand the specific and efficient interaction of aptamers to their cognate target, thus demonstrating the versatility and flexibility of nucleic acids.

Potential application of aptamers is an expanding field (for a review, see [3]). Since they can be easily synthesized and chemically modified, aptamers have emerged as attractive and feasible alternatives to small molecule and antibody-based therapies and diagnostic applications. Their features include high affinity and specificity, easy and large-scale synthesis by chemical methods, pharmaceutical versatility, and low immunogenicity [3]. The clinical value of aptamers has gained increasing relevance in the Virology field during last years. In this context, aptamers have been postulated not only as antiviral or biosensors, but also as tools for deciphering the molecular biology processes that govern the viral cycles of different viruses [4].

The isolation of aptamers directed against different protein targets of the hepatitis C virus (HCV) has been largely described [5]. The HCV belongs to the *Flaviviridae* family and is responsible for a worldwide health threat, infecting more than 180 million people (WHO data). Current treatments based on the combination of α-interferon with direct-acting antivirals (DAAs) targeting the viral protease NS3 are partially effective due to the appearance of resistant viral variants [6,7], a common problem for the control of viral infections. Thus, searching for novel drugs and targets is a major goal. In this context, a deep knowledge of the molecular biology of the virus is also a requisite for developing effective antiviral compounds.

**Figure 1:**
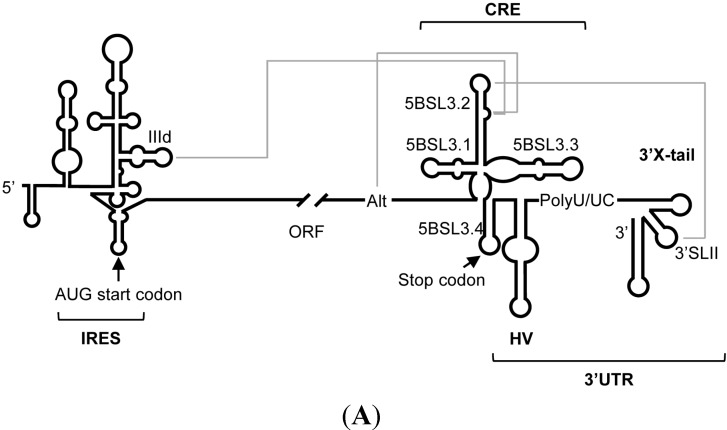

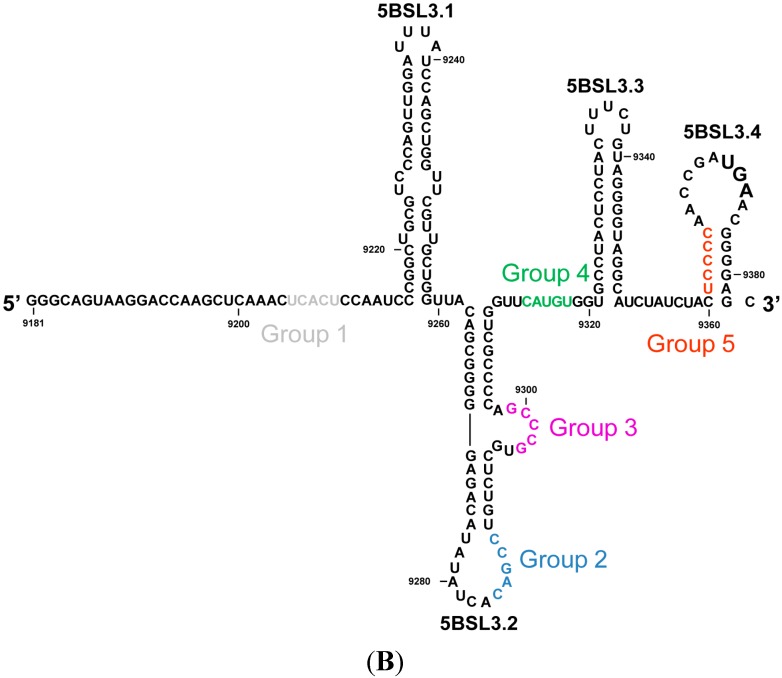
Structural organization of the HCV RNA genome. (**A**) Detailed diagram of the secondary structure proposed for the 5′ and 3′ ends of the HCV RNA including functional domains involved in the establishment of long-range RNA-RNA interactions. The minimal region required for the IRES activity is indicated. The architecture of the 3′ end of the viral genomic RNA is organized into two well-defined structural elements: the CRE region and the 3′X-tail. Translation start and stop codons are indicated by arrows. The grey solid lines identify long-distance RNA-RNA contacts involving the IRES, the CRE, the Alt, and the 3′X-tail regions. HV, hypervariable region; ORF, open reading frame; (**B**) The sequence and the secondary structure for the HCV CRE region. Nucleotides complementary to the consensus sequences of the selected aptamers are indicated by different colors according to the consensus group [34]. Numbers refer to the nucleotide positions of the HCV Con1 isolate (GenBank accession number AJ238799).

The HCV genome is a (+) polarity, single-stranded RNA (ssRNA) molecule of ~9600 nts, which encodes a single open reading frame (ORF) flanked by untranslated regions (UTRs) (Figure 1A) [8,9]. These UTRs contain highly conserved domains, both in sequence and in secondary structure, which are essential for viral replication, translation and infectivity [10,11,12,13,14,15,16]. Conserved functional RNA domains have also been identified within the coding region, such as the *cis*-acting replication element (CRE) in the 3′ end of the coding region (Figure 1) [17,18,19]. The CRE is defined by three stem-loops, 5BSL3.1, 5BSL3.2 and 5BSL3.3 [19] (Figure 1). While the role of the 5BSL3.1 and 5BSL3.3 domains is still unclear [20], the 5BSL3.2 domain (also named SL9266) has been largely studied. It consists of two G-C rich helices connected by an eight-base internal loop, and capped by a 12-base apical loop (Figure 1B) [19]. The preservation of both the sequence and the architecture of 5BSL3.2 is critical for efficient HCV replication [20,21] and the regulation of viral protein synthesis [22]. These functional features depend on the establishment of long-distant RNA-RNA interactions with other genomic RNA domains (Figure 1A). (i) The 5BSL3.2 apical loop is complementary to the apical loop of the 3′SLII within the 3′X-tail [21], their resulting kissing-loop interaction is required for efficient viral RNA synthesis in cell culture [21]; (ii) whereas the internal loop of the 5BSL3.2 domain may swap between two mutually exclusive contacts: one with the apical loop of the IIId subdomain of the IRES region [23]; and the second one with the Alt region placed upstream of the CRE [24]. Both interactions are equally probable and show dissociation constants in the same range [25]. Therefore, by switching from one contact to another, 5BSL3.2 could promote conformational rearrangements, not only in the directly involved residues and surrounding areas, but also, in an indirect manner, in the rest of the functional partners that compose this network [26,27,28]. In addition, the 5BSL3.2 domain interacts with viral and host protein factors [29,30,31]. All these data support the idea that the 5BSL3.2 domain is a key player in the maintenance of the proper balance between different stages of the infective cycle, which is a critical issue for viral fitness. Therefore, it can be considered as an interesting target for new therapeutic approaches against HCV. Extensive work is yet required in these areas.

We had previously reported the isolation of a collection of RNA aptamers targeting the CRE region of the HCV genome [32]. Some of these selected compounds were shown to interact with the 5BSL3.2 domain and to promote an inhibition of the viral replication of ~50% in cell culture [33]. The present study describes the functional characterization of a complete set of the previously isolated anti-CRE aptamers. From this screening, four molecules have emerged as efficient inhibitors of viral replication, leading to a robust decrease in viral RNA synthesis, close to a 95%. The selected aptamers contain sequence motifs targeting the highly conserved 5BSL3.2 domain and/or the stem-loop harboring the translation stop codon. They efficiently bind to the CRE region and promote changes in its functionality, either at the translational level or at the recruitment of the viral RNA-polymerase. Taken together, our findings support the use of the CRE as an efficient antiviral target and highlight the potential of aptamers as molecular tools for understanding the biological role of functional RNA domains in viral genomes.

## 2. Results and Discussion

### 2.1. Inhibition of HCV Replication by a Collection of RNA Aptamers Targeting the HCV CRE Region

It had been previously observed that a small set of aptamers targeting the HCV CRE region interfered with viral replication in cell culture [32,34], pointing to the CRE as a potential antiviral target. To go further on this hypothesis, a wide and representative set containing forty four different aptamers isolated for their binding ability to the CRE (see Table 1) [32] was assayed for their capacity to affect HCV replication. For that purpose, Huh-7 cells bearing a dicistronic selectable subgenomic replication construct derived from HCV-1b genotype (Huh-7 NS3-3′ET) [35,36] were transfected with the different aptamers independently. Total RNA was extracted 18 h post-transfection and the relative amount of the subgenomic HCV RNA was monitored by quantitative RT-PCR, as described in the Experimental Section. Transfection with a non-related small RNA molecule, RNA80, was used as a control experiment for data calibration [37,38].

**Table 1:** Aptamers used in this study.

Aptamer	SEQUENCE (5′→3′)	GROUP				
**P6-1**	CGUGGACGAGAGCUGGUAGUGUGUGGCGAU	1			4	
**P6-2**	GCUGCUGUUACGUACUAAGGUGCGGCGGGG					5
**P6-6**	CGGCUCUGGAUGGCGCUGUUUGUGUGUGGU				4	
**P6-8**	CAUUGUGCGACUGGGAGAGGGCGUGUCCG			3		
**P6-19**	CGUCCCGGCUGCGACAGGAUGGGGACAUGG		2			
**P6-20**	CAACGUGGCGAUGGCGUGUGUACGAUGUGG				4	
**P6-23**	CGUGUGCGCAGUGGGCAUCUGCGGACAGGG	1		3	4	
**P6-43**	GCAUCGGUGGGAAUUGCAGUGCCCGGCUGU		2	3		
**P6-44**	CGCGGCUUUGGGGACGUUAGCCAUCUGAUG					5
**P6-45**	CGUGUGUGCUGGCUAGUGGUGAGUCCGG	1			4	
**P6-50**	CGGAGGUUGUGUGGGGGACGUCUGUUGUGC		2		4	5
**P6-53**	CAGGUGGUGUUAGUUACGCGUAGGCGUGCC	1				
**P6-57**	GCGGCCUGCGAUCUGGAUGCUGCGUGGGCC			3		
**P6-64**	CCGAGGUGGCUGGGGACAGCAGGAGGAGCG	1	2			
**P6-76**	GGCAGCUCUAGAGGGGGCGUAAUCGGCUCG			3		
**P6-77**	GUGCUUGCGGUGUUGAGCCCAGCGGUAGUG	1				
**P6-78**	GGUACGGCAUGGCGCUACGGCUGGAUCGUG		2			
**P6-79**	GCUAUGGUGGCCUGGUCCGUCGGGGGGCCG	1				5
**P6-80**	CGCUAGUGUGGCGUGUUGCAGUAGGCAGAG				4	
**P6-81**	CAGGAUGAGUACUGGGCUCCUCGGCGUUGG			3		
**P6-82**	GUGUGUAUGCAUUGACGGACGACUGGCCGG				4	
**P6-83**	GGUGGAUUGGUGACCUUUGUGCUACGGGCA	1		3		
**P6-84**	CCCUGUGUUGGGCGGGCUACGUGUGUGGAG			3	4	
**P6-85**	GGGGCGUGUUCGGGACGCCUUGUACGAACG			3		
**P6-86**	CGGGCGUCGACUGAAGUUUGAGGUGGAAGGA	1		3		
**P6-87**	CGUGAUAGUUGUGCUGGCCGAUGGGUGGAC	1		3		
**P6-88**	CGCUGGUGGGUAGAGGUGUUUGUGUGCUGU	1		3	4	
**P6-89**	CGCCGUGCCAGCUCGGGACGGUGCGGCAGG			3		
**P6-91**	GUCGGCUGUUGACACGUGUAGUGUGGGUGG	1		3	4	
**P6-94**	CGCAGUGGAGGGCGAAUAAGAAUGUGACAG	1		3		
**P6-95**	CGGUGUGCGUGUGGGGACGCGUUCGUACAG				4	5
**P6-96**	CGUGUUACGGCUGUGCUGGGUACAUCGGUG		2	3		
**P6-98**	GAUGAGGCCUCGGUAGUGUGGACAGUGCAG				4	
**P6-99**	GUGGCCGUGUGGGCAACGGAACAUGCCGUG			3	4	
**P6-100**	CGUGUUACGGCUGUGCUGGGUACAUCGGUG		2			
**P6-101**	CGGUAACGUGGCCUUAGGGCAGGAGGCUG		2	3		
**P6-102**	CGGCACGAUGUGUCUACCGCGGUGGGGC	1			4	5
**P6-103**	GGUUGGACGUCGUCUGUGGGGGACUCGUGC		2			5
**P7-4**	GGCGUUGGUUUGUAUCGCGGCUUCGUGGGG					5
**P7-15**	CGUCCCAAUUGACACGUGGCAGGGGAGCCG					5
**P7-49**	CCGUGCGUGCAGUGGUUGGUCACGGCCUGG	1	2			
**P7-90**	CGGCCGUUGCUGGAGUGGUUGGCCGCAGUG	1	2			
**P7-93**	CAACUGCUCGUGUGUGGAGAGGGCGUGGCU			3	4	
**P7-97**	GACGUGUUCUGGCGUAUUGAGGGACGAUGG		2		4	

Sequence of the 30 nts-long variable RNA sequence of the selected aptamers, classified in groups according to common consensus sequences (colored). Shared nucleotides among different consensus motifs are depicted in italics, keeping the color corresponding to the consensus motif located in 5′.

**Figure 2:**
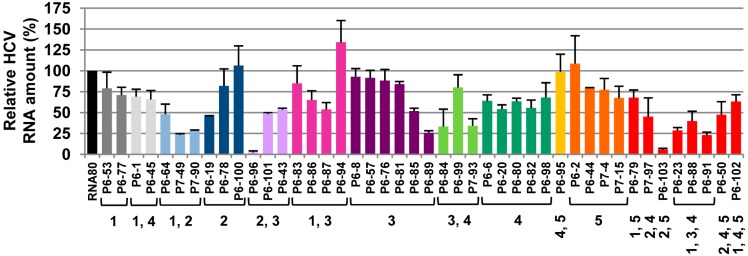
Inhibition of subgenomic HCV replicon replication in a hepatoma cell line. Huh-7 cells supporting the autonomous replication of subgenomic HCV replicons were transfected with 5 μg of the indicated aptamer. Viral RNA was isolated and quantified as described in the Experimental Section. The bar chart shows the (+) strand HCV RNA levels normalized to the value obtained with the RNA80, an 80 nts-long unrelated RNA used as an internal control. Values are the mean of at least four independent experiments.

The results showed that around 35% of the tested aptamers promoted a significant reduction (>50%) in the relative HCV RNA levels (Figure 2). Four out of them—P6-89, P6-96, P6-103 and P7-49—reached inhibition values higher than 75%. An additional 30% also interfered with viral RNA synthesis at certain degree (30%–50%). These data argue for considering the CRE region as a potential therapeutic target.

Interestingly, though most of the aptamers showed more than one theoretical interacting site within the CRE region (Table 1), around 62% of them contained in their sequence consensus motifs targeting the apical loop (group 2) or the internal loop (group 3) of the 5BSL3.2 domain. This observation suggests that the 5BSL3.2 domain may act as a recruiting platform for nucleic acids, which is in good agreement with its involvement in a network of long distance intragenomic RNA-RNA interactions. It is noteworthy that the aptamers promoting a reduction above 75% in the relative HCV RNA amount contain sequence motifs belonging to groups 2, 3, and/or 5 (Figure 2 and Table 1). The target of these groups maps in critical regions for viral propagation, such as the apical and internal loops of the 5BSL3.2 and the stem-loop containing the translation stop codon (Figure 1B) [32].

In summary, from this functional screening, four aptamers—P6-89, P6-96, P6-103, and P7-49—emerged as potential tools for HCV detection and inhibition.

### 2.2. Biochemical Analysis of the Selected Aptamers

Aptamers-mediated inhibition is directly related to their biochemical features, such as their three-dimensional folding and binding affinity to their target. Therefore, further efforts were focused on analyzing those properties.

**Figure 3:**
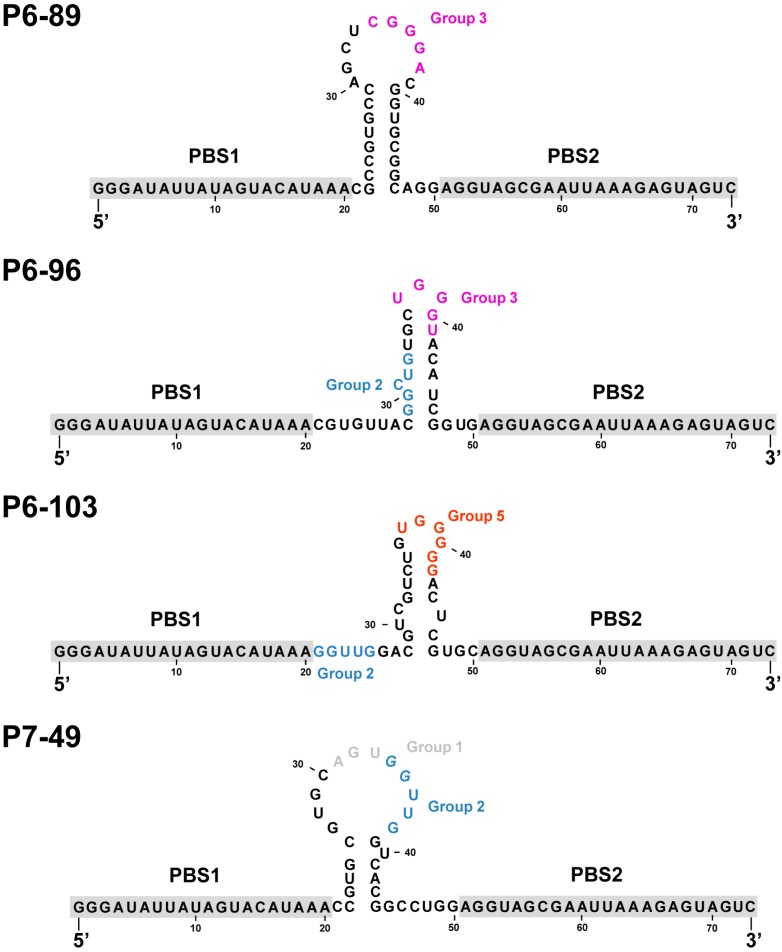
Proposed secondary structure of P6-86, P6-96, P6-103 and P7-49 as determined by TurboFold software. Theoretical nucleotide motifs involved in the interaction with the CRE are colored according to the group they belong to, as indicated in Figure 1B. The constant and common sequences for all the aptamers tested, PBS1 and PBS2, are highlighted in grey. PBS, primer binding site.

*In silico* structural analysis of the RNA molecules P6-89, P6-96, P6-103, and P7-49 was performed with the aim of identifying common structural motifs that could define a functional domain within the inhibitor RNA. The TurboFold tool was employed for that goal [39]. TurboFold is an iterative probabilistic method that uses a set of related sequences. It combines classical sequence comparison approaches and thermodynamic folding prediction to yield an estimation of the base pairing probabilities for each molecule. The use of this strategy reported a common secondary structure for the tested inhibitors (Figure 3), in which the constant sequences, corresponding to the ones used as primer binding site (PBS) during the selection process, appeared as single-stranded tails flanking the stem-loop containing the selected consensus motifs. These nucleotide motifs locate, at least partially, exposed in the apical loop (Figure 3). This folding gives the idea that the functional unit in the aptamer molecules is restricted to the stem-loop, which is used to efficiently interact with the target site in the CRE, in a similar way to that previously described for other regulatory RNA molecules [40]. This hypothesis prompted us to evaluate the binding ability of the aptamers to the CRE region.

**Figure 4:**
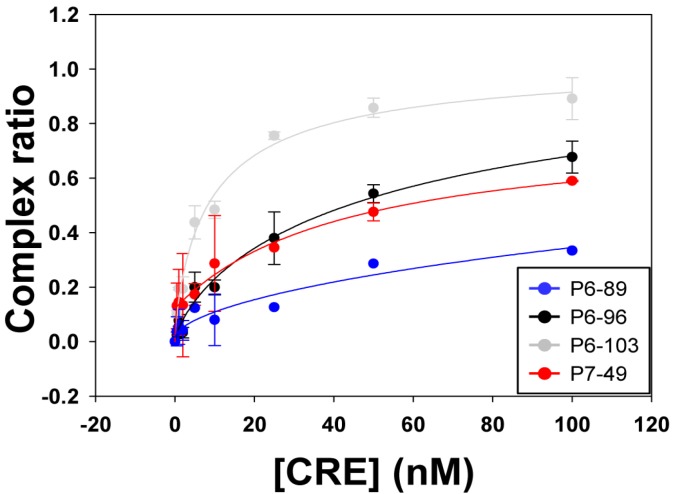
Binding assays of the aptamers P6-86, P6-96, P6-103, and P7-49 to the HCV CRE region. Graph shows the titration curve corresponding to the binding assays performed with the different aptamers under study. Internally ^32^P-labeled transcripts encompassing each of the tested aptamers were challenged with increasing amounts of their unlabeled interacting partner HCV-CRE_194_. Experiments were repeated at least three times and data were fitted to a non-linear equation for the calculation of the K_d_ value.

Binding affinity was analyzed by incubating a constant concentration of each ^32^P-internally labeled aptamer (~2 nM) with increasing amounts of the unlabeled construct CU [32], as detailed in the Experimental Section. This transcript CU bears the whole HCV CRE region from nucleotide 9181 (upstream of the 5BSL3.1 domain) plus the whole 3′UTR [28]. The titration curve showed differential interaction efficiency for the different aptamers under study (see Figure 4 and Table 2). Thus, aptamers containing the consensus motif from the group 2 (P6-96, P6-103 and P7-49) exhibited an efficient binding ability, with low K_d_ values and complex formation yields above 50% (Figure 4 and Table 2). The molecule P6-103 emerged as the most efficient interacting partner for the CRE, with a K_d_ value of 9.47 ± 3.49 nM and an extension complex formation of 1.04 ± 0.14. Interestingly, besides on bearing the group 2 consensus sequence, this aptamer contains the motif for group 5, which interacts with the translation stop codon. Finally, the variant P6-89, which targets the internal loop of the 5BSL3.2 domain (group 3), appeared as the less effective binder, with calculated K_d_ values in the range of low micromolar (see Figure 4 and Table 2).

These results suggest that the selected aptamers P6-96, P6-103, and P7-49 may exert their anti-HCV activity by directly interacting with the CRE region. Subsequent analyses were aimed at elucidating the mechanism of action of the aptamers under study.

**Table 2:** Binding constants for the selected aptamers.

Aptamer	K_d_ (nM) ± SD	B_max_ ± SD
**P6-89**	1706.34 ± 230.15	n.d.
**P6-96**	62.67 ± 0.74	1.15 ± 0.05
**P6-103**	9.47 ± 3.49	1.04 ± 0.14
**P7-49**	43.63 ± 16.22	1.07 ± 0.07

Values are the mean of three independent trials ± the standard deviation (SD). K_d_, dissociation constant; B_max_, final amplitude of the complex formation. n.d., non-determined.

### 2.3. Aptamers Targeting the 5BSL3.2 Domain Compete with the Recruitment of the Viral RNA-Dependent RNA Polymerase by the CRE Region

It has been described that the 5BSL3.2 domain binds to the viral NS5B protein [29,30]. Since some of the tested inhibitors harbor complementary sequence motifs to the 5BSL3.2 domain, it seemed reasonable to monitor whether the aptamers P6-89, P6-96, P6-103, and P7-49 could compete with the viral polymerase recruitment, as the replication inhibition mechanism. For that purpose, the aptamers were subjected to *in vitro* binding assays with the transcript HCV-CRE_194_ in the presence of the recombinant protein NS5BΔ21. Increasing concentrations of the aptamers under study were employed and the EC_50_ value was calculated.

**Figure 5:**
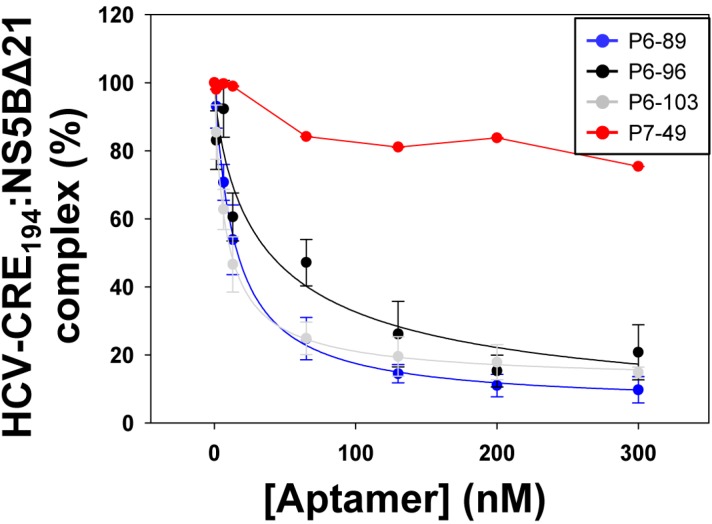
Competition of the interaction NS5BΔ21:CRE with the selected aptamers. The internally ^32^P-labeled HCV-CRE_194_ construct was incubated with a molar excess of the recombinant viral polymerase NS5BΔ21 and increasing concentrations of the aptamers under study, P6-86, P6-96, P6-103, and P7-49. Glycogen was used as a control of the competition reaction. Data were fitted to a non-linear equation to calculate the EC_50_ value. Values are the mean ± the standard deviation of four independent experiments.

The results showed that molecules P6-89, P6-96, and P6-103 efficiently interfered with the binding of the NS5B protein to the HCV-CRE_194_ transcript in a concentration-dependent manner with EC_50_ values in the nM range (Figure 5 and Table 3). Interestingly, the aptamer P7-49 barely showed a slight competitor activity (Figure 5). The addition of a non-related compound, such as glycogen, showed no binding inhibition activity (Figure 5), thus confirming the specificity of the observed competition.

**Table 3:** EC_50_ values for the competition of the interaction NS5BΔ21: CRE with the aptamers P6-89, P6-96, P6-103, and P7-49.

Aptamer	EC_50_ (nM) ^a^	HCV-CRE_194_:NS5BΔ21 Complex (%) ^b^
**P6-89**	14.59 ± 1.11	5.09 ± 2.04
**P6-96**	38.68 ± 5.63	0.00 ± 4.15
**P6-103**	8.57 ± 0.58	11.98 ± 1.46
**P7-49**	n.d.	75.37 ± 3.05

^a^ EC_50_ values were derived from the equation y = 100/(1 + 10^(LogEC50−x)^); ^b^ Data correspond to the highest concentration of inhibitor tested. Values are the mean of three independent assays ± the standard deviation (SD). n.d., non-determined.

These data demonstrate that the potential interaction of the aptamers P6-89, P6-96, and P6-103 with the CRE 5BSL3.2 domain interferes with the recruitment of the NS5B protein, which could be related to the observed anti-HCV effect.

### 2.4. Effect of the Aptamers on HCV Translation

**Figure 6:**
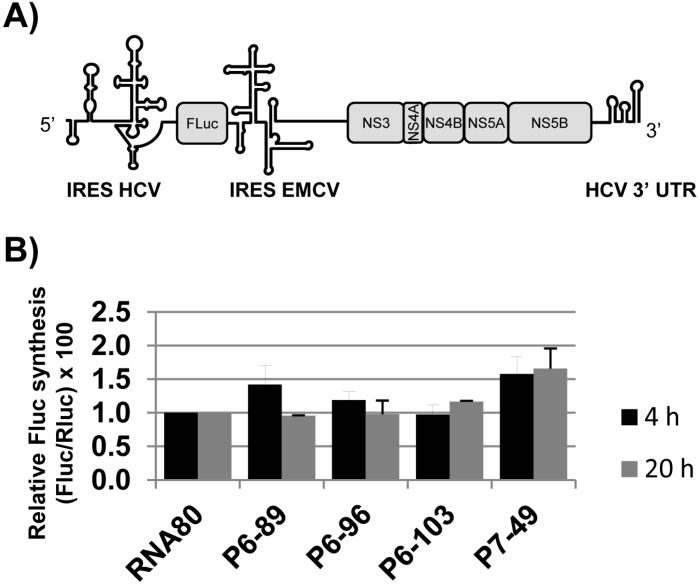
Effect on HCV translation of the aptamers P6-86, P6-96, P6-103, and P7-49 in Huh-7 cells. (**A**) Schematic diagram showing the genetic organization of the reporter dicistronic replicon Rep-FLuc used in this study; (**B**) Huh-7 cells were co-transfected by electroporation with the RNA aptamers and 5.2 μg of a mixture containing the transcripts Rep-FLuc and cap-RLuc. HCV IRES activity was measured as the activity of FLuc protein and referred to that obtained for RLuc. Luciferase activity in the control reactions with the non-related RNA80 is established as 100%. Data points are the mean of three independent experiments.

Aptamers P6-89, P6-96, P6-103, and P7-49 contain sequence motifs complementary to functional RNA domains that are involved in the regulation of HCV translation, such as the 5BSL3.2 [22] and the stem-loop containing the stop codon. This observation led us to study their role on viral protein synthesis in cell culture translation assays. With that aim, a mixture containing the so-called transcripts cap-RLuc, Rep-FLuc, and a 20-fold molar excess of the aptamers or the non-related RNA80 was used to transfect Huh-7.5 cells. The molecule Rep-FLuc bears a dicistronic reporter subgenomic replicon construct in which the *neo* selectable marker is substituted by the reporter gene *fluc* from the *Firefly* spp., whose translation is controlled by the HCV IRES region [41] (Figure 6A). This strategy allows to measuring the IRES activity in the early post-transfection period (4–20 h).

No effect was observed on protein synthesis for the inhibitors P6-96 and P6-103, suggesting that their potent inhibitory activity is mainly restricted to the replication step (Figure 6B). A significant increase in the FLuc activity was noted at early post-transfection times, when transfecting with the aptamer P6-89, but this effect was lately softened. Interestingly, the inhibitor P7-49 promoted a persistent enhancement (>50%, *p* < 0.05) in the IRES activity (Figure 6B), suggesting that this aptamer could target a functional region in the viral RNA involved in the regulation of IRES-dependent translation. No significant changes (<10%, *p* < 0.05) were detected for the cap-dependent translation, pointing to a specific effect on HCV protein synthesis.

Taken together, these data suggest that the tested inhibitors P6-96 and P6-103 specifically interfere with the HCV cycle mainly by blocking the replication step, while the molecules P6-89 and P7-49 exert a double role as HCV replication interfering agents and translational enhancers.

### 2.5. Discussion

Advances in clinical approaches for HCV detection and therapeutics are limited by the emergence of viral mutants. This phenomenon promotes the lack of effective antiviral sustained responses and false-negative tests. Therefore, it is desirable to search for novel targets that allow the development of efficient drugs and robust molecular diagnostic methodologies. Among the potential novel targets, the genomic RNA domains that play important roles in the consecution of essential viral processes are excellent candidates, which are worthy to be tested. In the context of both basic and applied research, the use of aptamers is recognized as a promising tool [5]. The present work describes the use of aptamers targeting highly conserved functional RNA domains of the HCV genome, provides important clues about their potential as antivirals and, more interestingly, highlights their use as molecular tools for understanding the functional role of the targeted RNA domains.

We had previously reported the isolation of a collection of RNA aptamers targeting the highly conserved CRE region of the HCV genome [32]. Interestingly, we found that two of these molecules directed against the functional 5BSL3.2 domain of the CRE were effective inhibitors of viral replication [34]. This work reports the extension of such findings to a complete collection of aptamers. Our data confirm the potential of using the 5BS3.2 domain as an antiviral target. They also support its function in viral translation and in the recruitment of the viral RNA-dependent RNA polymerase. Further, the results point to the stem-loop containing the stop codon as a novel efficient antiviral target and open a new field to investigate the role of this element in the consecution of the viral cycle.

The effectiveness of the CRE domain as a targeting region was evaluated. From the forty four molecules tested, 15 showed inhibition of HCV replication at rates higher than 50%. With the exception of variant P6-89, which targets the bulge of the 5BSL3.2, all of these efficient aptamers harbor sequence motifs complementary to the apical loop of the 5BSL3.2 domain. These results confirm the potential of the 5BSL3.2 as an anti-HCV target.

**Figure 7:**
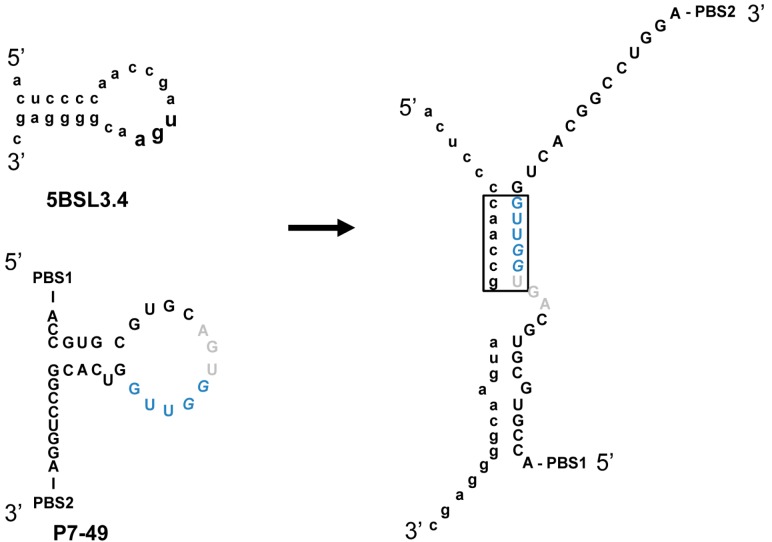
Theoretical model for the interaction between the 5BSL4 domain and the P7-49 RNA aptamer. CoFold and RNAup software were used to predict the nucleotides involved in the binding between the 5BSL4 stem-loop and the P7-49 aptamer. Residues proposed to initiate the kissing-loop interaction are boxed. Nucleotides belonging to groups 1 and 2 are colored as indicated in Figure 2. PBS, primer binding site.

Being the 5BSL3.2 a critical regulatory partner in the HCV IRES-dependent translation, some of the most efficient inhibitors of the viral replication were tested for their ability to affect the viral translation. Interestingly, two of the selected molecules, P6-89 and P7-49, promoted a significant increase in the IRES activity (Figure 6B). In the case of P6-89, this seems reasonable since it targets the internal loop of the 5BSL3.2. It has been previously reported that this motif acts as a negative partner of the HCV IRES function [22]. Therefore, it is likely that interfering with this activity can induce an enhancement of the IRES function. However, the role of P7-49 as an IRES-mediated translation inductor is surprising since this inhibitor theoretically interacts with the apical loop of the 5BSL3.2, which exerts its functionality mainly during viral replication [21]. It is plausible that P7-49 might interact with other essential domains of the CRE. In fact by using the folding software CoFold and RNAup, it was detected a major interacting site in the apical loop of the 5BSL3.4 domain, involving the stop codon (Figure 7). This newly predicted targeted motif differs slightly from that one previously defined for the group 5 in the initial sequence and clustering analysis [32]. This finding could be related to the enhancement of the HCV IRES-dependent translation observed for the P7-49 aptamer (Figure 6B). By modifying the conformation of the environment surrounding the stop codon, the ribosomal recycling could be enhanced to improve the IRES-dependent translation rate [42]. The theoretical interaction model proposes the establishment of a kissing-loop contact involving the apical loop of the 5BSL3.4 and the apical loop of P7-49. This kind of interaction is widely used in many biological systems to initiate and regulate a variety of molecular processes [40]. Further, it is widely exploited by RNA aptamers to efficiently recognize their cognate target [43]. The hypothesis of an alternative interacting site to the apical loop of the 5BSL3.2 element is also sustained by the observation that P7-49 is not able to compete with the binding of the NS5B protein (Figure 5). However, one cannot rule out the possibility that the aptamer might bind to both sites promoting different effects, the combination of which yields the measured inhibition of the viral replication. Neither can it be ruled out that this apparent contradictory effect on translation (enhancer) and replication (inhibitor) is not so, and the latter is a consequence of the former. Further investigation to understand whether the increase in viral translation might lead to the inhibition of HCV replication, or whether this resulting antiviral activity is due to an independent mechanism of the effect on translation should be carried out. Work in this area is currently being accomplished in our laboratory, since this idea opens new fields to evaluate the real role of the stem-loop containing the stop codon in the viral cycle, and for that purpose, P7-49 could be used as a novel molecular tool. In addition to the measured replication inhibition that reflects its potential as an anti-HCV molecule, the understanding of its mechanism of inhibition might yield a still unexplored anti-HCV strategy.

Finally, neither P6-96 nor P6-103 promoted any variation in the IRES activity (Figure 6B), which is consistent with the fact that both inhibitors bear complementary sequences to the apical loop of the 5BSL3.2 element. Accordingly, they efficiently compete with the recruitment of the viral RNA polymerase (Figure 5), which could be directly related to their mechanism of action [34].

In summary, this study provides evidences for the development of molecular tools based on aptamers with the aim of elucidating multiple aspects of the viral biology. From a clinical point of view, the design of alternative therapeutic strategies combining several antiviral molecules with different activities and specificities is also a feasible option that is gaining increasing support in recent years. Therefore, aptamers can be considered as multivalent partners in the Virology lab.

## 3. Experimental Section

### 3.1. Cell Lines and HCV Constructs

The human hepatoma cell line derivative Huh-7.5 are highly permissive for the initiation of HCV replication since they bear mutational inactivation of the retinoic acid-inducible gene I (RIG-I) [44]. Cell monolayers were maintained in high glucose Dulbecco’s modified Eagle medium (DMEM) (Gibco by Thermo Fisher Scientific, Waltham, MA, USA) with 10% heat-inactivated fetal bovine serum (Invitrogen by Thermo Fisher Scientific, Carlsbad, CA, USA), 1 mM sodium piruvate (Sigma-Aldrich Chemie, Steinheim, Germany) and 0.1 mM of non-essential aminoacids, NEAA, in MEM (Gibco by Thermo Fisher Scientific), at 37 °C in a 5% CO_2_ atmosphere.

Culture of the human hepatoma cell line Huh-7 NS3-3′ET supporting a subgenomic HCV replicon was carried out with DMEM-high glucose supplemented with 20% heat-inactivated fetal bovine serum, 1 mM sodium piruvate and 0.5 mg/mL of G-418 (Sigma-Aldrich Chemie) [38]. The subgenomic replicon carries the HCV IRES of genotype 1b, followed by the neomycin phosphotransferase gene (*neo*), the EMCV IRES, the coding sequence for non-structural HCV proteins (NS3-NS5) and the HCV 3′UTR [36].

### 3.2. DNA Templates and RNA Synthesis

DNA templates encoding the aptamer sequences were obtained by amplification as previously described [33]. The HCV-CRE_194_ DNA was obtained by *Sal*I digestion of the plasmid construct pUC18-T7HCV9181-9371, as reported [33]. DNA templates for the synthesis of the non-related RNA80 and cap-RLuc were constructed as specified in [37,45]. For the synthesis of the HCV subgenomic replicon system I_389_FLucNS3-3′ET, the plasmid pFK-I_389_FLucNS3-3′ET containing two cell-culture adaptive mutations in NS3 and one in NS5A, was restricted with *Spe*I. The RNA CU, bearing the CRE plus the whole HCV 3′UTR, was obtained as reported [28] for the binding assays.

The purified DNA molecules were used for *in vitro* RNA synthesis with the TranscriptAid T7 high yield transcription kit (Thermo Fisher Scientific), following the manufacturer instructions. Transcripts were purified as previously described [26]. RNA quality was monitored by UV spectrophotometry (NanoDrop by Thermo Scientific, Wilmington, DE, USA) and denaturing agarose-formaldehyde or urea-polyacrylamide gel electrophoresis.

### 3.3. Cell Transfection

Inhibitory activity of the RNA aptamers on HCV replication was assayed in the cell line Huh-7 NS3-3′ET as reported before [33,38]. Briefly, 48 h before transfection, ~90,000 cells were seeded onto 24-well plates in culture medium. An amount of 5 µg of each RNA aptamer or the non-related RNA80 was used for cell transfection using TransFectin™ lipid reagent (Bio-Rad, Hercules, CA, USA). Cells were harvested 18 h after transfection and subjected to subsequent analyses. Each transfection assay was performed in triplicate.

The effect of the aptamers on HCV translation was evaluated in Huh-7.5 cells. This cell line was transiently co-transfected with the reporter dicistronic subgenomic HCV replicon transcript, I_389_FLucNS3-3′ET, and the corresponding RNA aptamers or the RNA80. In addition, the reporter cap-RLuc transcript was included as internal transfection control. Cell were essentially electroporated as described [41], with minor variations. Briefly, monolayers of Huh-7.5 cells were trypsinized, counted and diluted to reach a concentration of 10^7^ cells/mL in Cytomix [46] supplemented with 2 mM ATP, 15 mM glutathione (Sigma-Aldrich Chemie) and 1.25% DMSO (Sigma-Aldrich Chemie). A mixture containing 5 µg of the subgenomic replicon construct, 200 ng of the cap-RLuc and 1 µg of each aptamer was incubated with the cell suspension on ice for 5 min. Electroporation was carried out at 1200 µF and 270 V in a cuvette with a gap width of 0.4 cm, using a BTX ECM 630 electroporation system (BTX ECM^®^ 630, Holliston, MA, USA). Cells were seeded onto 6-well plates with 4 mL of DMEM and 1.25% DMSO and harvested at 4 and 20 h post-transfection for *Firefly* and *Renilla* luciferase measurements.

### 3.4. Quantification of the HCV RNA Replicon

Intracellular relative HCV RNA amount was quantified by qRT-PCR as previously reported [38]. Briefly, total RNA was extracted with Trizol reagent (Invitrogen by Thermo Fisher Scientific) following the manufacturer’s instructions. Fifty ng of the purified RNA were then reverse transcribed with the High capacity cDNA reverse transcription kit (Applied Biosystems by Thermo Fisher Scientific) using random primers. A fraction of the extension reaction was employed for quantitative PCR with the SsoFast™ Evagreen^®^ supermix (Bio-Rad) and amplified over 40 cycles with specific oligonucleotides targeting the IRES region (C-149 and C-342) [47]. qRT-PCR of the mRNA encoding for the human glyceraldehyde-3-phosphate dehydrogenase (hGAPDH) was performed in parallel for relative quantification using the primers previously described (hGAPDH_Fw and hGAPDH_Rev) [48].

### 3.5. Binding Assays

RNA aptamer:CRE binding efficiency was monitored by incubation of 20 fmol of the internally labeled ^32^P aptamer with increasing amounts (0–1 pmol) of the unlabeled target RNA CU. RNA molecules were independently denatured by incubation at 95 °C during 2 min and then chilled on ice for 15 min. Reactions were initiated by mixing both RNA molecules in the presence of binding buffer (25 mM Tris-HCl pH 7.5, 300 mM KCl, 1 mM MgCl_2_). Complex formation proceeded during 15 min at 37 °C and the resulting products were loaded in 8% native polyacrylamide gels supplemented with MgCl_2_ 2 mM, in TBM buffer (45 mM Tris-HCl pH 8.3, 43 mM boric acid, 0.1 mM MgCl_2_). Electrophoresis proceeded at 12 mA and 4 °C during 3 h. Gels were then dried and further scanned using a Storm 820 PhosphorImager (GE Healthcare, Little Chalfont, Buckinghamshire, UK). Analysis was accomplished with the ImageQuant v5.2 software (Molecular Dynamics, Sunnyvale, CA, USA). Dissociation constants K_d_ were calculated with the Sigma Plot v8.0 software (Systat Software Inc., San José, CA, USA) and fitted to the equation y = (B_max_ × x)/(K_d_ + x), where y is the percentage of bound aptamer, B_max_ is the amplitude of the reaction, and x is the concentration of the substrate RNA CU [34].

### 3.6. Aptamer Competition with the Binding of the NS5BΔ21 to the HCV-CRE_194_

Recombinant viral RNA polymerase NS5BΔ21 was obtained as reported [34] and used for competition binding assays with the selected aptamers P6-89, P6-96, P6-103 and P7-49 [34]. RNA molecules were all independently denatured and folded as noted above before initiating the binding reactions. Fifty fmol of the ^32^P-internally-labelled HCV-CRE_194_ transcript were mixed with a 10-fold excess of the NS5BΔ21 in competition buffer (5 mM HEPES pH 7.9, 2 mM MgCl_2_, 25 mM KCl). This interaction was competed with increasing amounts of the selected aptamers or a non-related molecule, the glycogen (Ambion by Life Technologies). Binding reactions were performed at 37 °C during 30 min. They were then diluted in the competition buffer and applied to 0.45 µm nitrocellulose membranes (GE Healthcare). Filters were previously presoaked in the competition buffer and assembled in a dot blot apparatus (Bio-Dot Apparatus, Bio-Rad). Samples were filtered under vacuum. Membranes were dried, scanned, and analyzed as indicated above. The EC_50_ values were calculated from the equation y = y_max_/(1 + 10^(LogEC50−x)^), where y is the complex ratio, y_max_ is the maximum binding of the HCV-CRE_194_ RNA to the NS5BΔ21 protein, x is the aptamer concentration and EC_50_ the aptamer concentration that produces 50% of the maximum observed effect.

### 3.7. Luciferase Assays

*Firefly* and *Renilla* luciferase activities were determined with the Dual-Luciferase reporter assay system (Promega, Madison, WI, USA). All experiments were performed in triplicate and data are shown as normalized mean values ± standard deviation.
